# Bone mineral density as a prognostic marker in patients with non-small cell lung cancer undergoing neoadjuvant chemoimmunotherapy

**DOI:** 10.1186/s12957-025-04186-2

**Published:** 2026-01-08

**Authors:** Fengyi Zhou, Peile Li, Ji’an Zou, Weixuan Lei, Wei Han, Yun Gu, Yan Hu, Chao Zeng, Jina Li, Jieming Cao, Quanming Fei, Mengqi Shao, Junqi Yi, Zeyu Cheng, Li Wang, Yazhuo Liu, Wenliang Liu

**Affiliations:** 1 Department of Thoracic Surgery, The Second Xiangya Hospital, Central South University, No 139 Ren-Min Middle Road, Changsha, Hunan Province 410011 China; 2 Hunan Key Laboratory of Early Diagnosis and Precision Treatment of Lung Cancer, The Second Xiangya Hospital of Central South University, Changsha, China; 3 Department of Thoracic Surgery, Daping Hospital, Army Medical University, Chongqing, 400042 China

**Keywords:** Bone mineral density (BMD), Computed tomography (CT), Disease-free survival (DFS), Non-small cell lung cancer (NSCLC), Neoadjuvant chemoimmunotherapy (NICT)

## Abstract

**Background:**

Non-small cell lung cancer (NSCLC) is the most common lung cancer, and surgery is the primary curative treatment approach. Recently, neoadjuvant immunotherapy combined with chemotherapy (NICT) has become an important strategy. However, not all patients benefit, underscoring the need for reliable prognostic biomarkers. Bone mineral density (BMD) is associated with the prognosis of various cancers. This study explores the relationship between computed tomography (CT)-derived BMD and prognosis in NSCLC patients treated with NICT.

**Methods:**

101 stage IIA-IIIB NSCLC patients undergoing NICT and R0 resection surgery at our institution were included. Chest CT, highly correlated with dual-energy X-ray absorptiometry (DXA), was used to analyze the baseline and preoperative T10, T12, and L1 BMD. We analyzed disease-free survival (DFS) and overall survival (OS) through Kaplan-Meier survival curves, cox regression, and restricted cubic splines (RCS). Major pathologic response (MPR) and pathologic complete response (pCR) were compared through logistic regression. Subgroup analysis and sensitivity analysis were conducted to evaluate the accuracy of the results.

**Results:**

Median DFS was 22.58 months, and 14 patients experienced tumor recurrence or death. A significant decrease in BMD was observed at the T10, T12, and L1 vertebrae following NICT (*p* < 0.001). Patients with higher baseline and preoperative L1 BMD had significantly improved DFS (*p* = 0.002, *p* = 0.002). The prognostic value for BMD was confirmed through RCS analysis and multivariate Cox-regression analysis, as well as sensitivity analysis. Subgroup analysis revealed that the effect of L1 BMD on DFS showed significant interaction in age and MPR groups, suggesting age specific and treatment-response specific differences.

**Conclusion:**

Both baseline and preoperative L1 BMD are valuable, readily available, and independent prognostic biomarkers for DFS in NSCLC patients receiving NICT.

**Supplementary Information:**

The online version contains supplementary material available at 10.1186/s12957-025-04186-2.

## Highlights

**Key Findings**

• Baseline and preoperative L1 bone mineral density (BMD) independently predict disease-free survival (DFS) in non-small cell lung cancer (NSCLC) patients receiving neoadjuvant chemoimmunotherapy (NICT).

• Higher L1 BMD correlates with better DFS.

**What is known and what is new?**

• Known: NICT improves NSCLC outcomes, but response varies. BMD predicts prognosis in other cancers.

• New: First study linking computed tomography (CT)-derived L1 BMD to DFS in NSCLC under NICT. Low baseline and preoperative BMD are associated with poorer DFS; age and MPR influence BMD's prognostic value.

**What is the implication, and what should change now?**

• Use CT-derived BMD as a prognostic biomarker for personalized NSCLC treatment.

• Implement bone health strategies during NICT to improve outcomes.

• Investigate BMD-immunotherapy mechanisms and develop BMD-based prognostic models.

**Graphical abstract:**

**Supplementary Information:**

The online version contains supplementary material available at 10.1186/s12957-025-04186-2.

## Graphical abstract

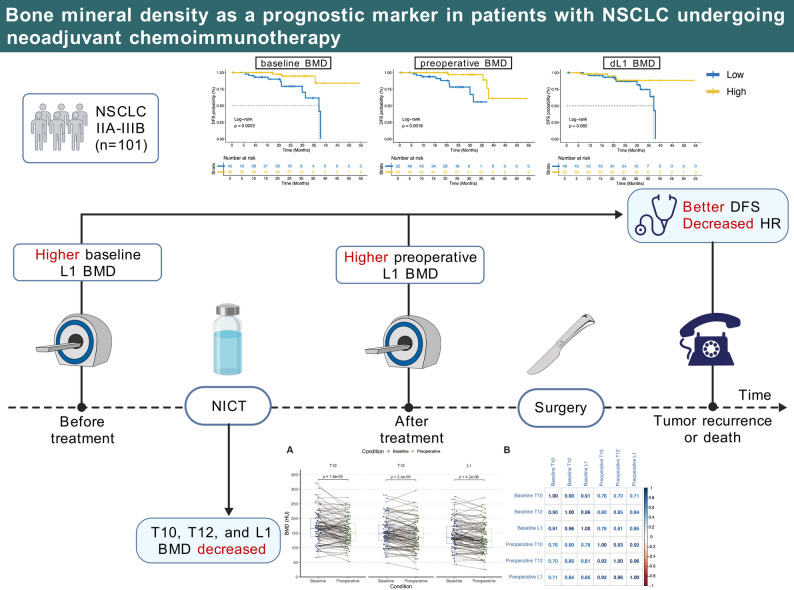

## Introduction

 Lung cancer ranks among the most common cancers and is the leading cause of cancer-related mortality globally. Non-small cell lung cancer (NSCLC) accounts for approximately 85% of all lung cancers [1]. Surgical resection is the primary curative option. However, the cancer recurrence rate remains high even after successful surgical resection, ranging from 30% to 55% [2, 3]. Recently, neoadjuvant immunotherapy combined with chemotherapy (NICT) has become an essential Strategy for resectable and potential resectable IIA-IIIB NSCLC. Clinical trials such as CheckMate-816, NADIM, KEYNOTE-671, AEGEAN, and CheckMate-77T have demonstrated satisfactory results for NICT and triple therapy in terms of major pathologic response (MPR), pathologic complete response (pCR), event-free survival, and overall survival (OS) [4–6]. However, due to considerable variability in treatment responses, some patients have not benefited from NICT. To improve the precision treatment and management of perioperative NSCLC, there is an urgent need to identify a convenient, inexpensive, and reliable biomarker for prognostic outcomes.

There is growing interest in the prognostic role of osteoporosis, defined by low bone mineral density (BMD), across diverse malignancies such as breast, colorectal, biliary tract, and lung cancer, etc [7–12]. Other studies have shown that in neoadjuvant therapy for breast cancer and colorectal cancer, patients experience varying degrees of bone mineral density loss [8, 13, 14]. In colorectal cancer research, the extent of bone density loss is closely associated with poor prognosis [8]. The association between low BMD and prognosis is hypothesized to stem from systemic factors like chronic inflammation and immune dysfunction associated with by osteoporosis and sarcopenia, combined with tumor-associated bone resorption that releases growth factors to promote tumor growth and metastasis [15, 16]. However, to our best knowledge, no study has systematically explored the relationship between baseline BMD, preoperative BMD, and the decrease in bone mineral density (dBMD) from baseline to preoperative with short-term and long-term outcomes in NSCLC patients receiving NICT.

Dual-energy X-ray absorptiometry (DXA) is the gold standard for assessing osteoporosis [17]. However, measuring the mean pixel density of the lumber vertebral trabeculae using computed tomography (CT) has become a recognized and convenient alternative method for assessing BMD [18, 19]. Studies have reported that CT-derived BMD values are highly correlated with z-score measured by DXA [20]. Since NSCLC patients typically undergo multiple chest CT scans through the treatment to assess their condition, dynamic monitoring of BMD changes becomes feasible. Therefore, CT-derived BMD demonstrates significant advantages in NSCLC patients receiving NICT and provides clinicians with additional, potentially prognostic information. Furthermore, as some chest CT scans cannot capture imaging data for L1 BMD, studies have shown that CT-derived lower thoracic vertebral BMD can also aid in diagnosing osteoporosis [21]. 

This study aimed to evaluate the association of CT-derived baseline and preoperative BMD, as well as dBMD, with short- and long-term outcomes in patients treated with NICT. The study’s primary endpoint is disease-free survival (DFS), while secondary endpoints include OS, MPR, and pCR. We analyzed DFS and OS through Kaplan-Meier survival curves, cox regression, and restricted cubic splines (RCS). MPR and pCR were compared through logistic regression. Subgroup analysis and sensitivity analysis were conducted to evaluate the accuracy of the results.

## Materials and methods

### Patients and ethical statement

We retrospectively reviewed data from patients with NSCLC who received NICT at The Second Xiangya Hospital, Central South University, from March 1, 2020, to December 30, 2023. The eligibility standards were as follows: (1) pathologically confirmed NSCLC; (2) completion of at least two cycles of chemotherapy combined with PD-1/PD-L1 inhibitors before surgery, followed by R0 resection; and (3) clinical staging of IIA-IIIB. The exclusion standards were as follows: (1) absence of baseline and preoperative non-contrast chest CT scans or poor-quality CT images; (2) incomplete clinical, pathological, or follow-up data; (3) concurrent advanced malignancies; and (4) coexistence of autoimmune diseases. All patients underwent standardized preoperative staging assessments, including tumor biopsy, radiological imaging, and invasive mediastinal lymph node staging procedures (endobronchial ultrasound or mediastinoscopy). Tumor staging was determined according to the eighth edition of the TNM staging system.

This study was approved by the institutional ethics committee (Approval Number: LYF20250012) and conducted strictly with the Declaration of Helsinki and reported with STROBE guidelines. Informed consent was obtained from the patients.

### Bone mineral density measurement and assessment

The measurement of BMD was based on chest CT images obtained within 30 days before treatment and surgery. The most recent scan was selected for analysis if multiple CT scan records were available for a patient. All CT scans were performed using a 128-slice CT scanner (Somatom Perspective 128, Siemens, Germany) with consistent parameters: tube voltage of 120 kVp, automated tube current modulation, and a slice thickness of 1 mm. Non-contrast CT images with a slice thickness of 1 mm were extracted from the Picture Archiving and Communication System for analysis. All images were acquired in the supine position at full inspiration. The measurement of BMD was independently conducted by two thoracic surgeons blinded to the patients’ clinical data.

**Fig. 1:**
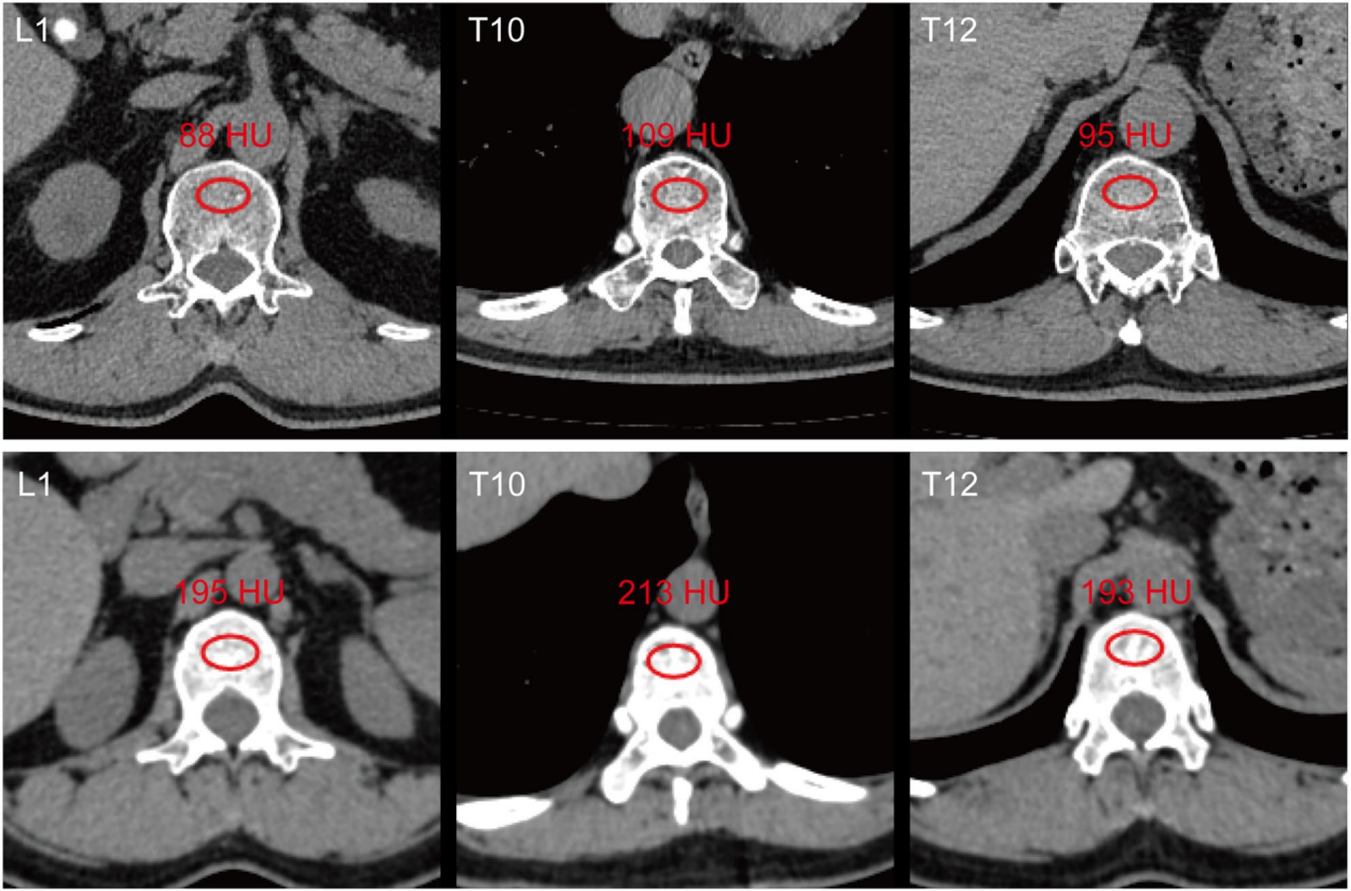
Examples of CT-derived Bone Mineral Density (BMD) Assessment. Representative transverse (axial) CT images at the L1, T10, and T12 vertebral levels showing patients with varying Hounsfield unit (HU) values. The red circular Regions of Interest (ROI) demonstrate the standard placement for trabecular attenuation measurement, positioned in the core of the vertebral body while strictly avoiding the cortical bone and posterior venous plexus. The mean HU value within each ROI is displayed

Regions of interest (ROI) were selected at the core of the T10, T12, and L1 vertebral bodies, avoiding cortical bone and vertebral veins while maximizing the inclusion of trabecular bone, as shown in (Fig. 1) [11, 12, 18]. The average pixel density -- Hounsfield unit (HU) within the selected ROI was calculated. Each ROI was drawn three times to reduce measurement error, and the mean value was taken. If the BMD difference between the two surgeons exceeded 30 HU, a third observer redrew the ROI and recalculated the values. The inter-observer consistency for baseline and preoperative L1 BMD were 0.996 and 0.998, respectively.

The optimal cutoff value for BMD was determined using the *surv_cutpoint* function in the R package *survminer* (version 0.4.9) and validated using *x-tile* software (version 3.6.1) [22]. All patients were divided into high and low BMD groups based on the BMD values and the degree of BMD reduction. Additionally, referring to relevant literature, age-adjusted standard BMD was calculated using the following formulas [23]:

$$\mathrm{Male}:\;\mathrm{BMD}\;(\mathrm{HU})\;=\;308.82\;-\;2.49\;\times\;\mathrm{Age}$$

$$\mathrm{Female}:\;\mathrm{BMD}\;(\mathrm{HU})\;=\;311.84\;-\;2.41\;\times\;\mathrm{Age}$$

### Study variables

(1) Clinicopathological Characteristics: Clinical demographic information includes age (< 65 years and ≥ 65 years), gender, smoking status (former or current and never), and body mass index (BMI, < 25 kg/m² and ≥ 25 kg/m²). Tumor clinical features encompass clinical stage (IIA-B, IIIA, IIIB; due to only five patients with stage IIA, it was combined with stage IIB into IIA-B), clinical T stage (T1-T4), clinical N stage (N0, N1, N2-3; due to only two patients with stage N3, it was combined with stage N2 into N2-3), histological subtype (squamous cell carcinoma and non-squamous carcinoma), tumor location (left and right), PD-L1 expression levels (Tumor Proportion Score: <1%, 1–50%, > 50%, not known), and lesion size (mm). Information related to NICT includes chemotherapy regimens (paclitaxel/carboplatin, paclitaxel/cisplatin, pemetrexed/carboplatin, pemetrexed/cisplatin), specific types of immune checkpoint inhibitors (anti-PD-L1: atezolizumab, envolimab, sugilimumab; anti-PD-1: camrelizumab, nivolumab, pabrolizumab, sintilimab, tislelizumab), dosage (2, 3, and 4 cycles), CT scan interval (days), and treatment interval (> 6 weeks and ≤ 6 weeks) [24].

(2) Plasma Biomarkers Potentially Related to BMD: Baseline and preoperative plasma calcium concentrations (mmol/L), the albumin-to-globulin ratio (A/G).

(3) CT-derived bone mineral density: The primary analysis focuses on the baseline BMD, preoperative BMD, and the dBMD from baseline to preoperative levels at L1. Based on cut-off values, patients are categorized into high and low groups. BMD at T10 and T12 vertebrae are also measured simultaneously to evaluate the accuracy of CT-determined BMD in reflecting bone status and for sensitivity analysis.

(4) Clinical Outcomes: Long-term outcomes include DFS: The time from R0 surgery to tumor recurrence or death. OS: The time from R0 surgery to death from any cause. Short-term outcomes include Major Pathological Response (MPR): ≤10% of tumor cells remain viable in the postoperative tumor specimen. Pathological Complete Response (pCR): no viable tumor cells are present in the postoperative tumor specimen.

### Statistical analysis

Continuous variables with normal distribution are expressed as mean ± standard deviation (SD) and analyzed using independent or paired t-tests. Non-normally distributed continuous variables are presented as medians with interquartile ranges and analyzed using the Mann-Whitney U test. Categorical variables are presented as frequencies and percentages, with intergroup differences assessed using the chi-square test (χ²). Correlation analysis between variables is conducted using Pearson’s correlation coefficient.

The optimal cut-off value for bone mineral density is determined using the surv_cutpoint function in the R package *survminer (version 0.4.9)* and validated using *X-tile software (version 3.6.1)*. In survival analysis, Kaplan-Meier is used to generate survival curves, with intergroup differences assessed using the log-rank test. Variables with *p* < 0.2 in univariate Cox regression are selected, and those with potential severe multicollinearity are excluded before inclusion in the multivariable Cox regression model for further analysis. Restricted cubic splines (RCS) are applied to assess nonlinear relationships and optimize the nodes. In subgroup analysis and sensitivity analyses, the HU values of T10, T12, and L1 BMD are standardized (per SD unit) and analyzed using univariate Cox regression. For analyses of MPR and pCR, univariate logistic regression is used to identify variables with *p* < 0.2, and those with potential severe multicollinearity are excluded before inclusion in the multivariable logistic regression model for analysis.

All statistical analyses are conducted using R software (version 4.4.1), and a two-sided *p* < 0.05 is considered statistically significant.

## Results

### Patient characteristics

Table 1 lists the patient characteristics included in this study. We retrospectively analyzed 101 patients with stage IIA-IIIB NSCLC who had undergone NICT. After R0 surgery, 75 patients (74.26%) achieved MPR, 51 (68%) achieved pCR. Baseline characteristics included age, gender, smoking status, BMI, tumor clinical features, NICT-related information, and plasma biomarkers.

**Table 1:** Baseline characteristics of patients based on MPR

Characteristics		Total (*N* = 101)	Non-MPR (*N* = 26)	MPR (*N* = 75)	*p*
Age (years)	Mean ± SD (Range)	59.8 ± 7.2 (41–82)	62.6 ± 8.6 (48–82)	58.8 ± 6.4 (41–72)	**0.020**
	< 65	71 (70.3%)	13 (50%)	58 (77.3%)	**0.017**
	≥ 65	30 (29.7%)	13 (50%)	17 (22.7%)	
Gender	Female	6 (5.9%)	1 (3.8%)	5 (6.7%)	0.966
	Male	95 (94.1%)	25 (96.2%)	70 (93.3%)	
Smoke	Former or current	71 (70.3%)	17 (65.4%)	54 (72%)	0.699
	Never	30 (29.7%)	9 (34.6%)	21 (28%)	
BMI (Kg/m²)	< 25	54 (53.5%)	13 (50%)	41 (54.7%)	0.855
	≥ 25	47 (46.5%)	13 (50%)	34 (45.3%)	
Clinical stage	IIA-B	24 (23.8%)	7 (26.9%)	17 (22.7%)	0.493
	IIIA	41 (40.6%)	8 (30.8%)	33 (44%)	
	IIIB	36 (35.6%)	11 (42.3%)	25 (33.3%)	
cT	T1	10 (9.9%)	3 (11.5%)	7 (9.3%)	0.941
	T2	36 (35.6%)	9 (34.6%)	27 (36%)	
	T3	28 (27.7%)	8 (30.8%)	20 (26.7%)	
	T4	27 (26.7%)	6 (23.1%)	21 (28%)	
cN	N0	14 (13.9%)	4 (15.4%)	10 (13.3%)	0.952
	N1	25 (24.8%)	6 (23.1%)	19 (25.3%)	
	N2-3	62 (61.4%)	16 (61.5%)	46 (61.3%)	
Histologic subtype	Non-squamous	23 (22.8%)	10 (38.5%)	13 (17.3%)	0.052
	Squamous	78 (77.2%)	16 (61.5%)	62 (82.7%)	
Location	Left	36 (35.6%)	11 (42.3%)	25 (33.3%)	0.558
	Right	65 (64.4%)	15 (57.7%)	50 (66.7%)	
PD-L1 (TPS)	< 1%	10 (9.9%)	3 (11.5%)	7 (9.3%)	0.889
	1–50%	59 (58.4%)	16 (61.5%)	43 (57.3%)	
	> 50%	29 (28.7%)	6 (23.1%)	23 (30.7%)	
	Not known	3 (3.0%)	1 (3.8%)	2 (2.7%)	
Chemotherapy	Paclitaxel + Carboplatin	75 (74.3%)	15 (57.7%)	60 (80%)	**0.014**
	Paclitaxel + Cisplatin	9 (8.9%)	2 (7.7%)	7 (9.3%)	
	Pemetrexed + Carboplatin	15 (14.9%)	7 (26.9%)	8 (10.7%)	
	Pemetrexed + Cisplatin	2 (2.0%)	2 (7.7%)	0 (0%)	
Immunotherapy	Atezolizumab	1 (1.0%)	0 (0%)	1 (1.3%)	0.591
	Camrelizumab	4 (4.0%)	2 (7.7%)	2 (2.7%)	
	Envolimab	2 (2.0%)	1 (3.8%)	1 (1.3%)	
	Nivolumab	7 (6.9%)	3 (11.5%)	4 (5.3%)	
	Pabrolizumab	37 (36.6%)	11 (42.3%)	26 (34.7%)	
	Sintilimab	9 (8.9%)	2 (7.7%)	7 (9.3%)	
	Sugilimumab	2 (2.0%)	0 (0%)	2 (2.7%)	
	Tislelizumab	39 (38.6%)	7 (26.9%)	32 (42.7%)	
Dosage	2	25 (24.8%)	7 (26.9%)	18 (24%)	0.957
	3	40 (39.6%)	10 (38.5%)	30 (40%)	
	4	36 (35.6%)	9 (34.6%)	27 (36%)	
CT scan interval (days)	Mean ± SD (Range)	111.5 ± 42.5 (35–237)	113.2 ± 43.0 (37–193)	111.0 ± 42.6 (35–237)	0.824
Treatment interval (weeks)	> 6	55 (54.5%)	16 (61.5%)	39 (52%)	0.54
	≤ 6	46 (45.5%)	10 (38.5%)	36 (48%)	
Lesion size (mm)	Mean ± SD	25.5 ± 14.1	28.9 ± 14.1	24.4 ± 14.1	0.162
Baseline serum Ca (mmol/L)	Mean ± SD	2.3 ± 0.2	2.3 ± 0.3	2.3 ± 0.1	0.453
Baseline A/G	Mean ± SD	1.4 ± 0.3	1.4 ± 0.2	1.4 ± 0.3	0.663
Preoperative serum Ca (mmol/L)	Mean ± SD	2.2 ± 0.3	2.2 ± 0.3	2.2 ± 0.3	0.608
Preoperative A/G	Mean ± SD	1.5 ± 0.3	1.5 ± 0.3	1.5 ± 0.2	0.435
pCR	No	50 (49.5%)	26 (100%)	24 (32%)	**< 0.001**
	Yes	51 (50.5%)	0 (0%)	51 (68%)	
pN	N0	90 (89.1%)	20 (76.9%)	70 (93.3%)	**0.027**
	N1	2 (2.0%)	0 (0%)	2 (2.7%)	
	N2	6 (5.9%)	4 (15.4%)	2 (2.7%)	
	N1 + N2	3 (3.0%)	2 (7.7%)	1 (1.3%)	
OS (months)	Median (IQR)	28.85 (19.15–36.18)	27.18 (19.15–32.92)	29.87 (18.69–36.52)	0.514
DFS (months)	Median (IQR)	22.58 (14.92–22.63)	20.82 (14.05–28.22)	25.27 (16.14–31.19)	0.193
Baseline L1 BMD (HU)	Mean ± SD	145.1 ± 46.6	133.2 ± 55.1	149.3 ± 42.9	0.131
Preoperative L1 BMD (HU)	Mean ± SD	133.0 ± 47.9	121.0 ± 50.6	137.2 ± 46.6	0.139
dL1 BMD (HU)	Mean ± SD	12.1 ± 26.3	12.2 ± 31.8	12.1 ± 24.3	0.98
Baseline L1 BMD	Low	45 (44.6%)	14 (53.8%)	31 (41.3%)	0.38
	High	56 (55.4%)	12 (46.2%)	44 (58.7%)	
Preoperative L1 BMD	Low	52 (51.5%)	17 (65.4%)	35 (46.7%)	0.156
	High	49 (48.5%)	9 (34.6%)	40 (53.3%)	
dL1 BMD	Low	48 (47.5%)	12 (46.2%)	36 (48%)	1
	High	53 (52.5%)	14 (53.8%)	39 (52%)	

*A/G* Albumin to globulin ratio, *BMD* Bone mineral density, *HU* Hounsfield unit, *MPR* Major pathologic response, *pCR* Pathologic complete response, *pN* Pathologic N stage, *SD* Standard deviation, *dL1 BMD* Decrease in L1 bone mineral density, *TPS* Tumor proportion score Values in bold indicate statistical significance (P < 0.05)

Among the patients, 30 (29.7%) were aged ≥ 65 years, 95 (94.1%) were male, and 71 (70.3%) were former or current smokers. Regarding pathological types, 78 patients (77.2%) had squamous cell carcinoma, while 23 patients (22.8%) had other types of NSCLC. The long-term outcomes included DFS and OS, with median follow-up times of 22.58 months and 28.85 months, respectively.

We found that patients achieved MPR were more likely to be younger, having squamous cancer, receiving paclitaxel + cisplatin chemotherapy, with pathological N0 stage. Surgical details and other postoperative pathological information are presented in Supplemental Tables 1 and Supplemental Table 2.

The baseline, preoperative and decrease in L1 bone mineral density (dL1 BMD) were 145.1 ± 46.6, 133.0 ± 47.9, and 12.1 ± 26.3 HU, respectively. The baseline and preoperative L1 BMD (149.3 ± 42.9 and 137.2 ± 46.6 HU) in the MPR group were numerically higher than those in the non-MPR group (133.2 ± 55.1 and 121.0 ± 50.6 HU, *p* = 0.131 and 0.139, respectively). The dL1 BMD between the two groups didn’t show difference (*p* = 0.98). T10 and T12 BMD and age-adjust BMD are shown in Supplemental Table 3.

### Changes in BMD and correlation analysis

The median time interval between the baseline and preoperative CT scans was 107 days (Range: 35–237 days; Mean ± SD: 111.5 ± 42.5 days). Correlation analysis indicated a weak positive correlation between the scan interval and the decrease in L1 BMD (*r* = 0.251, *p* = 0.011), suggesting that a longer treatment interval contributes to greater bone loss. However, univariate Cox regression analysis showed that the scan interval was not significantly associated with DFS (HR = 1.00, 95% CI: 0.98–1.01, *p* = 0.757).

**Fig. 2:**
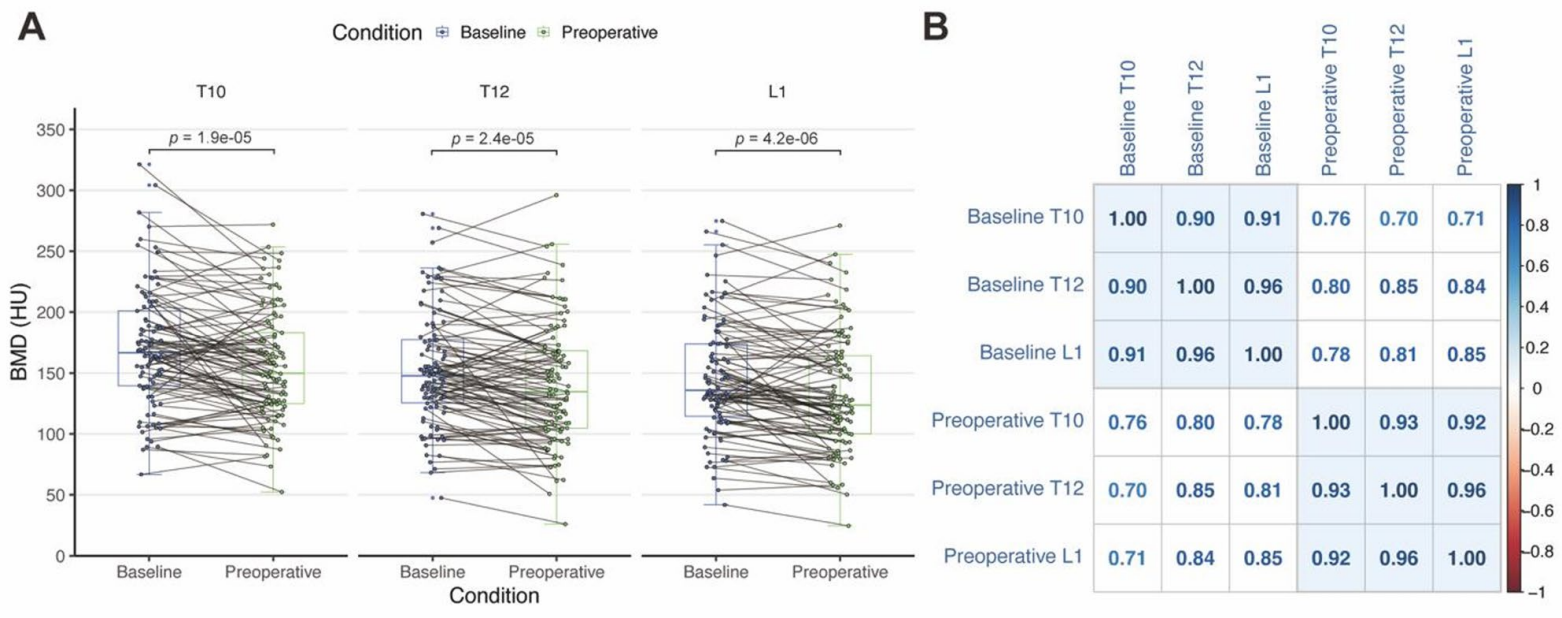
Changes in vertebral bone mineral density and correlation analysis

Figure 2 illustrates the changes and correlations among baseline and preoperative T10, T12, and L1 BMD. After NICT treatment, T10, T12, and L1 BMD significantly decreased (*p* < 0.001) **(**Fig. 2A**)**, suggesting NICT may lead to a loss in BMD, resulting in osteoporosis, which is consistent with previous research findings [8, 13, 14]. The intra-period correlations of BMD (i.e., Baseline-to-Baseline and Preoperative-to-Preoperative) among different vertebrae at baseline and preoperatively ranged from 0.90 to 0.96 (*p* < 0.001) **(**Fig. 2B**)**, suggesting that CT-derived L1 BMD can effectively reflect the bone status of patients, providing a reliable metric for clinical assessment.

### Association of L1 BMD with DFS

**Fig. 3:**
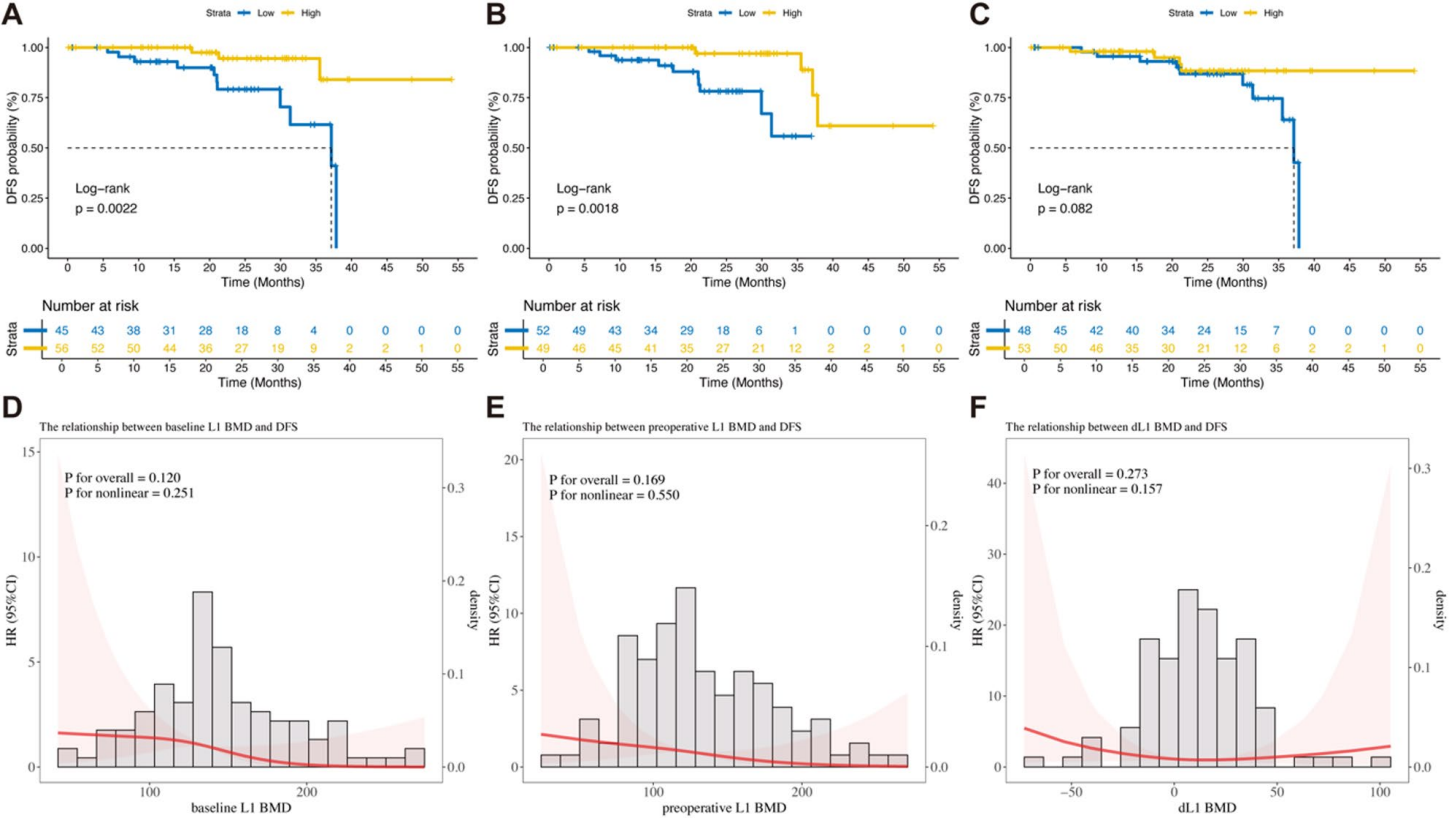
Association of L1 BMD with DFS. **A**-**C** Kaplan-Meier curves of the baseline/preoperative/dL1 BMD. The optimal cutoff values are 132 HU, 124.2 HU, and 9.73 HU, respectively. **D**-**F** Non-linear relationship between baseline/preoperative/dL1 BMD and DFS using RCS curve with 3 knots

Figure 3 illustrates the impact of baseline and preoperative L1 BMD and dL1 BMD on DFS. The optimal cut-off values for baseline/preoperative L1 and dL1 BMD were 132 HU, 124.2 HU, and 9.73 HU, respectively.

Patients with higher baseline and preoperative L1 BMD had significantly longer DFS compared to the lower BMD groups (*p* = 0.002 and 0.002, respectively). Although patients with higher dL1 BMD also had relatively longer DFS, this did not reach statistical significance (*p* = 0.082) (Figs. 3A-C). The nonlinear regression relationship between baseline/preoperative L1 BMD and dL1 BMD with DFS were analyzed by RCS curve (Figs. 3D-F). The results indicate that the HR for DFS decreases as baseline/preoperative L1 BMD increases. For dL1 BMD, the HR initially decreases and then slightly increases, with a turning point around 30 HU.

The impact of baseline and preoperative L1 BMD and dL1 BMD on OS were shown in Supplemental Fig. 1. Patients with higher baseline and preoperative L1 BMD had longer OS than those in the lower BMD group (*p* = 0.2, *p* = 0.038), while no significant difference in OS was observed between high and low dL1 BMD groups (*p* = 0.99). Kaplan-Meier survival curves for DFS and OS based on age-adjusted BMD groups are presented in Supplemental Fig. 2.

Table 2 presents the results of Cox regression analyses for various characteristics associated with DFS. In the univariable analysis, squamous cell carcinoma, lesion size, and high baseline/preoperative L1 BMD were significantly associated with DFS. The multivariable analysis included factors including age, pathological subtype, tumor size, MPR, preoperative L1 BMD, and dL1 BMD. The results indicated that tumor size (HR = 1.06, *p* = 0.013), high preoperative L1 BMD (HR = 0.09, *p* = 0.012), and high dL1 BMD (HR = 0.26, *p* = 0.036) were significantly associated with longer DFS.

**Table 2:** Uni-variable and muti-variable Cox regression analysis on DFS

Characteristics		HR (uni-variable)	HR (multi-variable)^*2^
Age (years)	< 65		
	≥ 65	2.56 (0.82–8.01, *p* = 0.106)	1.26 (0.30–5.31, *p* = 0.755)
Gender	Female		
	Male	1.09 (0.14–8.54, *p* = 0.933)	
Smoke	Former or current		
	Never	1.50 (0.44–5.09, *p* = 0.514)	
BMI (Kg/m2)	< 25		
	≥ 25	1.17 (0.40–3.43, *p* = 0.781)	
Clinical stage	IIA-B		
	IIIA	3.00 (0.36–24.80, *p* = 0.308)	
	IIIB	2.76 (0.32–23.50, *p* = 0.352)	
cT	T1		
	T2	0.28 (0.04–2.00.04.00, *p* = 0.205)	
	T3	1.13 (0.20–6.45, *p* = 0.889)	
	T4	0.91 (0.18–4.56, *p* = 0.911)	
cN	N0		
	N1	2.46 (0.27–22.20, *p* = 0.422)	
	N2-3	1.31 (0.16–10.90, *p* = 0.805)	
Histologic subtype	Non-squamous		
	Squamous	0.23 (0.07–0.72, p = **0.012**)	0.69 (0.13–3.68, *p* = 0.660)
Location	Left		
	Right	1.92 (0.59–6.21, *p* = 0.276)	
PD-L1 (TPS)	——^*1^		
Chemotherapy	——^*1^		
Immunotherapy	——^*1^		
Dosage	2		
	3	1.10 (0.29–4.17, *p* = 0.884)	
	4	0.98 (0.26–3.70, *p* = 0.972)	
CT scan interval (days)		1.00 (0.98–1.01, *p* = 0.757)	
Treatment interval (weeks)	> 6		
	≤ 6	1.21 (0.42–3.49, *p* = 0.724)	
Lesion size (mm)		1.06 (1.02–1.09, p = **0.002**)	1.06 (1.01–1.1, p = **0.013**)
Baseline serum Ca (mmol/L)		2.31 (0.12–46.00.12.00, *p* = 0.584)	
Baseline A/G		0.53 (0.07–4.10, *p* = 0.546)	
Preoperative serum Ca (mmol/L)		0.75 (0.25–2.25, *p* = 0.602)	
Preoperative A/G		1.82 (0.22–14.90, *p* = 0.577)	
MPR	No		
	Yes	0.38 (0.12–1.22, *p* = 0.105)	1.61(0.27–9.51, *p* = 0.597)
pCR	No		
	Yes	0.98 (0.34–2.81, *p* = 0.973)	
pN	——^*1^		
Baseline L1 BMD (HU)		0.98 (0.97–1.00.97.00, p = **0.015**)	
Preoperative L1 BMD (HU)		0.99 (0.97–1.00.97.00, p = **0.042**)	
dL1 BMD (HU)		0.99 (0.97–1.01, *p* = 0.466)	
Baseline L1 BMD	Low		
	High	0.17 (0.05–0.61, p = **0.007**)	
Preoperative L1 BMD	Low		
	High	0.11 (0.02–0.55, p = **0.007**)	0.09 (0.01–0.59, p = **0.012**)
	Low		
dL1 BMD	High	0.36 (0.11–1.19, *p* = 0.094)	0.26 (0.07–0.92, p = **0.036**)

^*1^Due to the small sample size of some subgroups, PD-L1 (TPS), chemotherapy, immunotherapy, and pN were excluded from the Cox regression analysis

^*2^In the univariate Cox regression analysis, variables with a p-value < 0.2 were included in the multivariate analysis. To avoid the impact of multicollinearity, Baseline L1 BMD (HU), Preoperative L1 BMD (HU), and Baseline L1 BMD, which were highly correlated with Preoperative L1 BMD, were manually excluded from the multivariate analysis

*A/G* Albumin to globulin ratio, *BMD* Bone mineral density, *HU* Hounsfield unit, *MPR* Major pathologic response, *pCR* Pathologic complete response, *pN* Pathologic N stage, *SD* Standard deviation, *dL1 BMD* Decrease in L1 bone mineral density, *TPS* Tumor proportion score Values in bold indicate statistical significance (P < 0.05)

### Subgroup analysis and sensitivity analysis

**Fig. 4:**
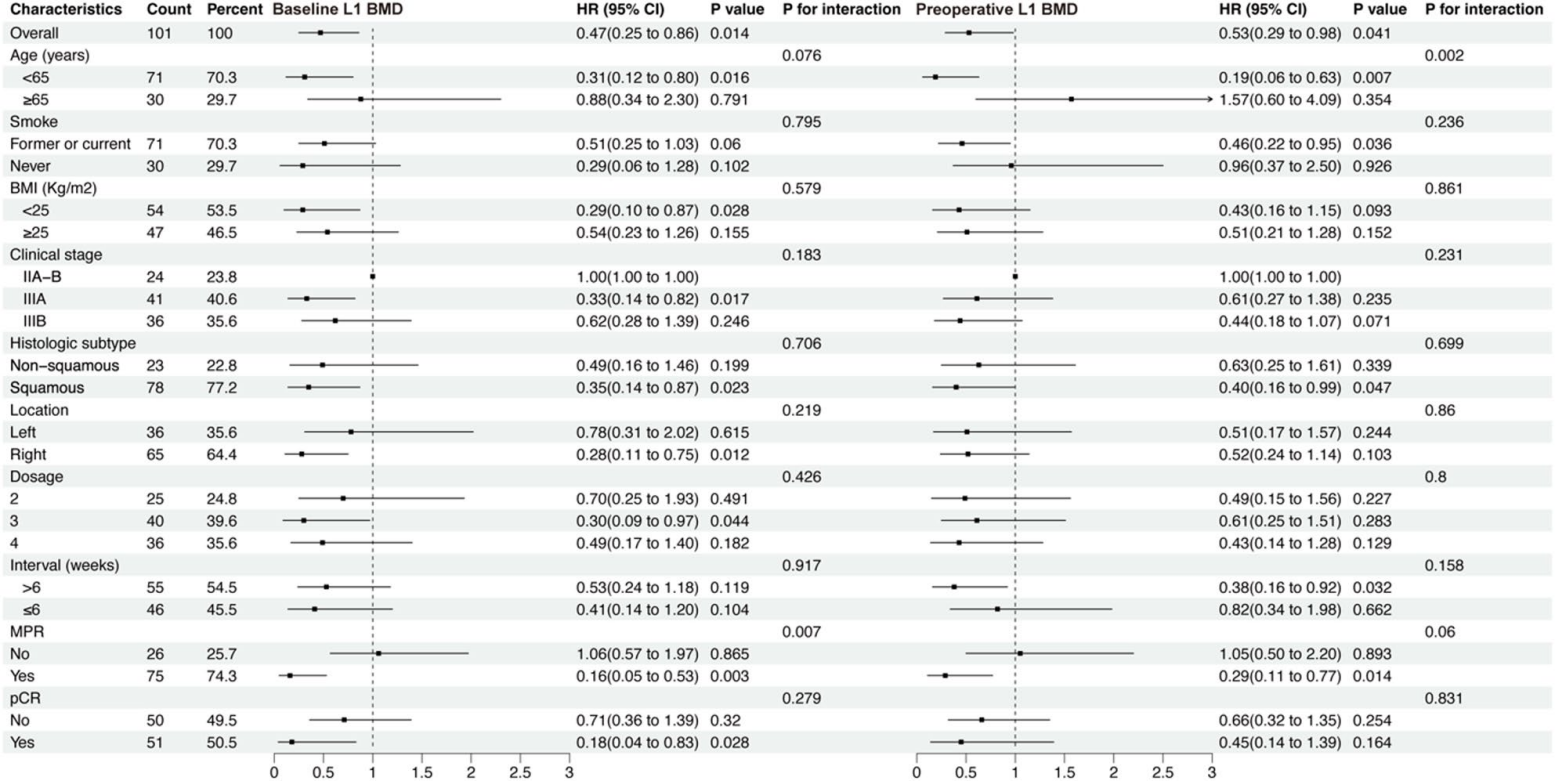
Subgroup Cox regression analysis of L1 BMD (per SD change) on DFS

Figure 4 present the univariate Cox subgroup analysis results for baseline and preoperative L1 BMD (per SD). Increased L1 BMD consistently acts as a protective factor for DFS in most of the subgroups. However, the effect of baseline and preoperative L1 BMD on DFS showed significant interaction in age groups (p for interaction = 0.076,0.002) and MPR groups (p for interaction = 0.007,0.06), suggesting age and treatment response specific differences. Specifically, L1 BMD provides more substantial protective effects for younger patients and patients who achieved MPR. Detailed results are provided in Supplemental Tables 4 and Supplemental Table 5.

**Fig. 5:**
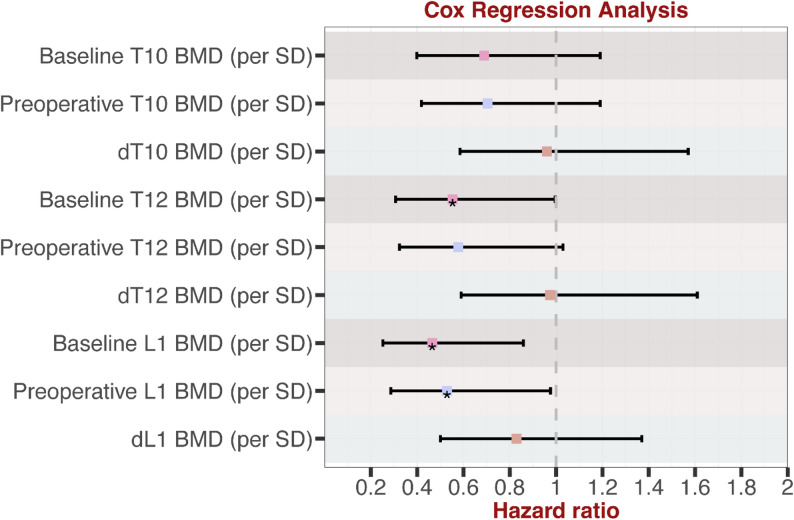
Sensitivity analysis of BMD (per SD change) on DFS using Cox regression analysis

The univariate Cox regression results for baseline/preoperative BMD and dBMD (per SD) across different vertebrae with DFS were presented in Fig. 5 and Supplemental Table 6. Increased T10, T12, and L1 BMD were associated with prolonged DFS (HR < 1), demonstrating a protective effect. Baseline T12, L1 BMD and preoperative L1 BMD achieved statistical significance (*p* < 0.05). These findings further support the positive correlation between higher BMD and longer DFS. It also suggests that L1 BMD is more sensitive as a prognostic biomarker.

### Association of L1 BMD with MPR

**Fig. 6:**
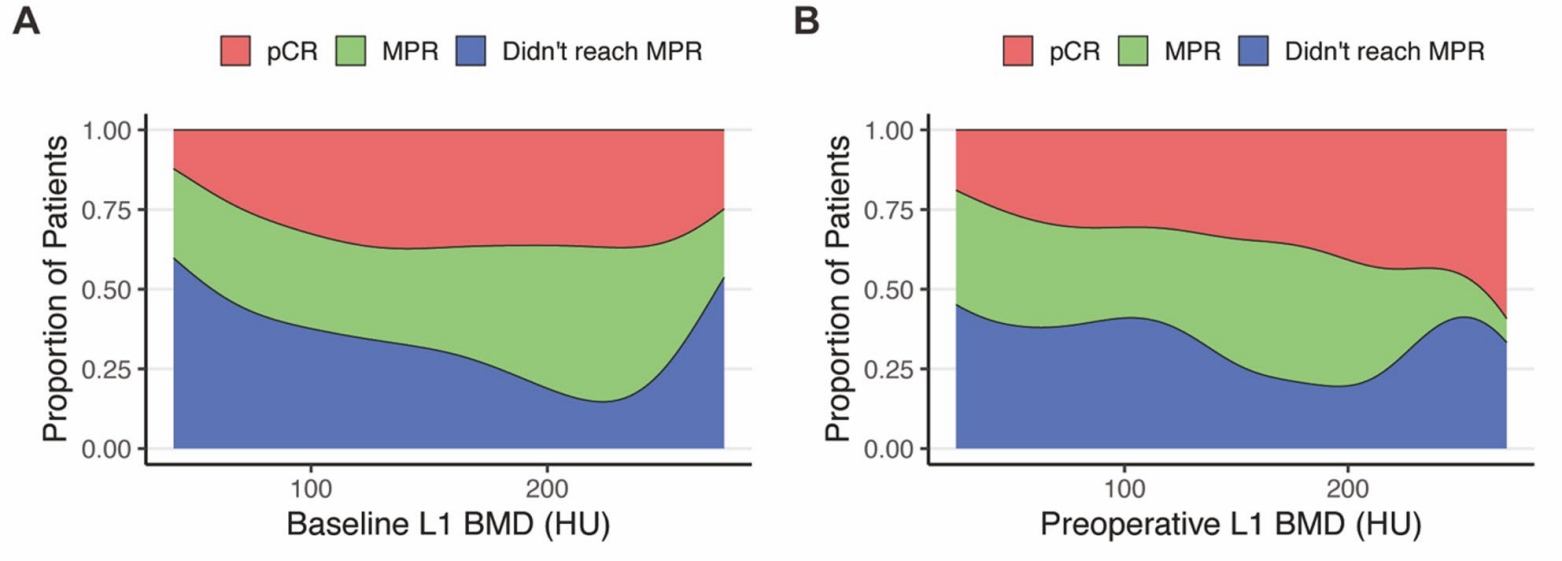
Relationship between L1 BMD and pathological state

Table 3 presents the results of logistic regression analyses for MPR. Patients ≥ 65 years and squamous cell carcinoma showed a lower MPR probability (uni: OR = 0.29, *p* = 0.010; multi: OR = 0.30, *p* = 0.020) and a near-significant association, respectively (uni: OR = 2.98, *p* = 0.031; multi: OR = 2.77, *p* = 0.067). L1 BMD was not significantly associated with either MPR or pCR (Supplemental Table 6). Fig. 6 presents the relationship between baseline/preoperative L1 BMD with pathological state. As baseline L1 BMD increased, the proportion of patients achieving MPR rose, while pCR initially increased and then plateaued. However, higher L1 BMD levels were associated with a significant rise in patients not achieving MPR. Preoperative L1 BMD was positively correlated with a higher pCR rate.

**Table 3:** Uni-variable and multi-variable logistic regression on MPR

Characteristics		OR (uni-variable)	OR (multi-variable)^*2^
Age (years)	< 65		
	≥ 65	0.29 (0.11–0.75, p = **0.010**)	0.30 (0.11–0.82, p = **0.020**)
Gender	Female		
	Male	0.56 (0.06–5.03, *p* = 0.605)	
Smoke	Former or current		
	Never	0.73 (0.28–1.90, *p* = 0.525)	
BMI (Kg/m²)	< 25		
	≥ 25	0.83 (0.34–2.03, *p* = 0.681)	
Clinical stage	IIA-B		
	IIIA	1.70 (0.53–5.48, *p* = 0.375)	
	IIIB	0.94 (0.30–2.90, *p* = 0.908)	
cT	T1		
	T2	1.29 (0.27–6.05, *p* = 0.750)	
	T3	1.07 (0.22–5.21, *p* = 0.932)	
	T4	1.50 (0.29–7.65, *p* = 0.626)	
cN	N0		
	N1	1.27 (0.29–5.56, *p* = 0.754)	
	N2-3	1.15 (0.32–4.18, *p* = 0.832)	
Histologic subtype	Non-squamous		
	Squamous	2.98 (1.11–8.03, p = **0.031**)	2.77 (0.93–8.25, *p* = 0.067)
Location	Left		
	Right	1.47 (0.59–3.66, *p* = 0.412)	
PD-L1 (TPS)	< 1%		
	> 50%	1.64 (0.32–8.33, *p* = 0.549)	
	1–50%	1.15 (0.27–5.01, *p* = 0.851)	
	Not known	0.86 (0.05–13.48, *p* = 0.913)	
Chemotherapy	——^*1^		
Immunotherapy	——^*1^		
Dosage	2		
	3	1.17 (0.38–3.61, *p* = 0.789)	
	4	1.17 (0.37–3.70, *p* = 0.793)	
Treatment interval (weeks)	> 6 week		
	≤ 6 week	1.48 (0.59–3.67, *p* = 0.401)	
Lesion size (mm)	Mean ± SD	0.98 (0.95–1.01, *p* = 0.164)	0.98 (0.95–1.02, *p* = 0.300)
pN	——^*1^		
Baseline serum Ca (mmol/L)	Mean ± SD	3.82 (0.32–46.27, *p* = 0.292)	
Baseline A/G	Mean ± SD	1.49 (0.25–8.70, *p* = 0.659)	
Preoperative serum Ca (mmol/L)	Mean ± SD	0.68 (0.16–2.95, *p* = 0.605)	
Preoperative A/G	Mean ± SD	2.06 (0.34–12.42, *p* = 0.431)	
Baseline L1 BMD (HU)	Mean ± SD	1.01 (1.00–1.02.00.02, *p* = 0.134)	
Preoperative L1 BMD (HU)	Mean ± SD	1.01 (1.00–1.02.00.02, *p* = 0.141)	
dL1 BMD (HU)	Mean ± SD	1.00 (0.98–1.02, *p* = 0.979)	
Baseline L1 BMD	Low		
	High	1.66 (0.67–4.06, *p* = 0.271)	
Preoperative L1 BMD	Low		
	High	2.16 (0.85–5.45, *p* = 0.104)	1.35 (0.48–3.77, *p* = 0.567)
dL1 BMD	Low		
	High	0.93 (0.38–2.27, *p* = 0.871)	

^*1^Due to the small sample size of some subgroups, chemotherapy, immunotherapy, and pN were excluded from the logistic regression analysis

^*2^In the univariate logistic regression analysis, variables with a p-value < 0.2 were included in the multivariate analysis. To avoid the impact of multicollinearity, Baseline L1 BMD (HU) and Preoperative L1 BMD (HU), which were highly correlated with Preoperative L1 BMD, were manually excluded from the multivariate analysis

*A/G* Albumin to globulin ratio, *BMD* Bone mineral density, *HU* Hounsfield unit, *MPR* Major pathologic response, *pN* Pathologic N stage, *SD* Standard deviation, *dL1 BMD* Decrease in L1 bone mineral density, *TPS* Tumor proportion score Values in bold indicate statistical significance (P < 0.05)

## Discussion

Responses to NICT vary substantially among patients with NSCLC, highlighting considerable interindividual heterogeneity in treatment efficacy [4–6] Identifying biomarkers to predict NICT outcomes could optimize treatment strategies and improve clinical management. Prognostic biomarkers like PD-L1 [25], tumor mutational burden [26], and microsatellite instability [27] assist in personalized treatment. Recently, peripheral blood markers such as circulating tumor DNA [6], neutrophil-to-lymphocyte ratio (NLR) [28], and T-cell subtypes [29] come to prominence. Based on preoperative non-contrast CT [30], contrast-enhanced CT [31], and positron emission tomography–computed tomography [32, 33], predictive models for MPR have been developed. However, information provided by CT still requires further exploration to establish more comprehensive, convenient, and reliable prognostic biomarkers. Osteoporosis and sarcopenia are two key manifestations of skeletal muscle dysfunction, both can be assessed using CT [34]. While DXA is the gold standard for assessing osteoporosis [17], it is not routinely performed in NSCLC patients, as it is not required for staging or surgical evaluation. In contrast, CT, as a routine examination before and after NICT, is widely available in perioperative BMD assessment. The frequent chest CT scans during treatment facilitate dynamic monitoring of BMD changes. Recent studies have demonstrated a high correlation between CT-measured BMD and DXA [20]. Based on these advantages, this study utilized CT to assess BMD.

This study is the first to demonstrate that low baseline and preoperative L1 BMD are independent prognostic factors for DFS in NSCLC patients undergoing NICT and R0 surgical resection. Additionally, we observed a significant decrease in vertebral BMD after treatment. To our knowledge, this is the first study to analyze the relationship between baseline, preoperative vertebral BMD, its dynamic changes, and prognosis in NSCLC patients treated with NICT.

Our findings are consistent with previous studies on the prognosis of advanced NSCLC patients receiving immune checkpoint inhibitors and those undergoing surgery for brain metastases [11, 12]. In this study, patients with low baseline and preoperative L1 BMD (132 HU/124.2 HU) has significantly lower DFS. Cox regression analyses, RCS analysis, and subgroup analyses further confirmed the relationship. Similar trends were also observed in the T10 and T12 vertebrae. Subgroup analysis further revealed that the effect of L1 BMD on DFS showed significant interaction in age and MPR groups, suggesting age specific and treatment-response specific differences. For patients younger than 65 and those achieving MPR, low BMD was significantly associated with lower DFS. This could be attributed to the fact that younger individuals typically do not experience age-related bone loss, making their prognosis more sensitive to variations in BMD. For those achieving MPR, recurrence may be more closely linked to overall health status which can be reflected by BMD. Although high dL1 BMD showed near-statistical significance for longer DFS in the Kaplan-Meier curve (*p* = 0.082), consistent results were not observed in the RCS or sensitivity analyses. Regarding the decrease in BMD, although multivariate analysis suggested a potential association with DFS, the univariate analysis did not reach statistical significance. This suggests that baseline physiological reserve (reflected by static BMD) may be a more robust prognostic indicator than the dynamic change in BMD during short-term neoadjuvant therapy. Furthermore, the lack of OS differences between dL1 BMD groups implies that the rapid bone loss observed during NICT may not immediately translate to long-term survival capability compared to the patient’s initial skeletal health. No direct correlation between BMD and MPR/pCR was observed. This suggests that pathological response and survival outcomes are driven by distinct mechanisms in the context of NICT. Pathological response (MPR/pCR) is largely determined by tumor-intrinsic factors (e.g., tumor immunogenicity, mutation burden), whereas DFS is a composite outcome influenced by both tumor control and the host’s systemic resilience (e.g., skeletal health, nutritional status, and immune function). Therefore, BMD serves as a prognostic marker for the host’s physiological reserve rather than a predictive marker for tumor sensitivity to chemoimmunotherapy.

BMD indicates the mineral content (mainly calcium) in bones. Studies have shown that low BMD is an independent marker of poor prognosis in cancers such as breast, colorectal, biliary duct, and lung cancer [7–12]. Although the mechanisms linking low BMD to poor DFS remain incompletely understood, several hypotheses may explain this association: First, the interaction between osteoporosis and sarcopenia may lead to chronic inflammation and a weakened immune system, thereby accelerating tumor recurrence [15]. Second, vitamin D deficiency, which is strongly associated with osteoporosis, has been reported to be associated with recurrence in various cancers, suggesting that osteopenia reflects vitamin D deficiency [35–37]. Third, osteoporosis is often associated with aging, malnutrition, and reduced quality of life, all closely linked to tumor recurrence [37]. In addition, reduced physical activity, exercise, and a sedentary lifestyle often accompany osteoporosis and sarcopenia, compounding the decline in overall health status and contributing to an increased risk of tumor recurrence [38]. Tumor-secreted cytokines such as parathyroid hormone-related protein can enhance the expression of receptor activator of nuclear factor kappa-B ligand, activating osteoclasts and inducing the receptor activator of nuclear factor kappa-B ligand/receptor activator of nuclear factor kappa-B pathway, thereby promoting tumor stemness and metastasis [39]. Furthermore, tumor-associated bone resorption releases various growth factors, such as transforming growth factor-beta, insulin-like growth factor, and platelet-derived growth factor, from the mineralized bone matrix, which can stimulate tumor growth [16]. 

Despite these findings, for patients whose surgical indications are determined based on clinical staging, relying solely on preoperative BMD assessment should not rule out the possibility of surgery. Therefore, we recommend measuring BMD at both stages (before and after treatment) to help identify high-risk patients. This may contribute to providing more precise perioperative treatment strategies for high-risk NSCLC patients or implementing targeted interventions during recovery. Meanwhile, biomarkers such as BMD can help stratify the prognosis of NSCLC patients receiving NICT. Rather than predicting therapeutic efficacy, BMD aids in identifying high-risk patients with poor physiological reserve. These patients, despite receiving standard neoadjuvant therapy, remain at higher risk for recurrence and may require intensified postoperative surveillance or supportive care interventions. In the future, it may even be possible to train specialized artificial intelligence models that incorporate BMD as a parameter into clinical evaluations.

This study has several limitations. First, this was a single-center, retrospective study with a relatively small sample size, predominantly comprising males with squamous cell carcinoma. Second, selection bias may exist because it is a retrospective study. Third, the relatively short follow-up period may affect the observation of DFS and OS. Therefore, future large-scale multicenter prospective studies with more extended follow-up periods are needed to validate our findings.

## Conclusion

Both baseline and preoperative L1 BMD are valuable, readily obtainable, and independent prognostic biomarkers for DFS in patients with NSCLC undergoing NICT. Assessing CT-derived BMD before and during treatment provide valuable prognostic information, offering high-risk NSCLC patients more precise perioperative treatment strategies.

## Supplementary Information

Supplementary Material 1.

## Data Availability

The datasets of the current study can be accessed from the corresponding author upon reasonable request.

## Abbreviations

BMD: Bone mineral density
CT: Computed tomography
dBMD: Decrease in bone mineral density
dL1 BMD: Decrease in L1 bone mineral density
DFS: Disease-free survival
DXA: Dual-energy X-ray absorptiometry
HU: Hounsfield unit
MPR: Major pathologic response
NICT: Neoadjuvant immunotherapy combined with chemotherapy
NSCLC: Non-small cell lung cancer
OS: Overall survival
pCR: Pathologic complete response
RCS: Restricted cubic splines
ROI: Regions of interest
SD: Standard deviation
TPS: Tumor proportion score

## References

[1] Bray F, Laversanne M, Sung H, et al. Global cancer statistics 2022: GLOBOCAN estimates of incidence and mortality worldwide for 36 cancers in 185 countries. CA Cancer J Clin. 2024;74(3):229–63. 10.3322/caac.21834. 38572751 10.3322/caac.21834

[2] Nemesure B, Albano D, Bilfinger T. Lung cancer recurrence and mortality outcomes over a 10-year period using a multidisciplinary team approach. Cancer Epidemiol. 2020;68:101804. 10.1016/j.canep.2020.101804. 32896806 10.1016/j.canep.2020.101804

[3] Peters S, Weder W, Dafni U, et al. Lungscape: resected non-small-cell lung cancer outcome by clinical and pathological parameters. J Thorac Oncol. 2014;9(11):1675–84. 10.1097/JTO.0000000000000320. 25436801 10.1097/JTO.0000000000000320

[4] Duan J, Tan F, Bi N, et al. Expert consensus on perioperative treatment for non-small cell lung cancer. Transl Lung Cancer Res. 2022;11(7):1247–67. 10.21037/tlcr-22-527. 35958323 10.21037/tlcr-22-527 PMC9359944

[5] Forde PM, Spicer J, Lu S, et al. Neoadjuvant nivolumab plus chemotherapy in resectable lung cancer. N Engl J Med. 2022;386(21):1973–85. 10.1056/NEJMoa2202170. 35403841 10.1056/NEJMoa2202170 PMC9844511

[6] Provencio M, Nadal E, Insa A, et al. Neoadjuvant chemotherapy and nivolumab in resectable non-small-cell lung cancer (NADIM): an open-label, multicentre, single-arm, phase 2 trial. Lancet Oncol. 2020;21(11):1413–22. 10.1016/S1470-2045(20)30453-8. 32979984 10.1016/S1470-2045(20)30453-8

[7] Omori S, Ijichi H, Wakasugi A, et al. Association between preoperative osteopenia and prognosis in breast cancer patients. Anticancer Res. 2024;44(6):2671–9. 10.21873/anticanres.17074. 38821581 10.21873/anticanres.17074

[8] Dang W, Wu S, Liu X, et al. Association between quantitative CT body composition analysis and prognosis in cetuximab-based first-line treatment for advanced colorectal cancer patients. BMC Cancer. 2024;24(1):1579. 10.1186/s12885-024-13338-8. 39725871 10.1186/s12885-024-13338-8 PMC11670449

[9] Heinrichs L, Fluegen G, Loosen SH, et al. Bone mineral density as a prognostic marker in patients with biliary tract cancer undergoing surgery. BJC Reports. 2024;2(1):72. 10.1038/s44276-024-00094-2. 39323978 10.1038/s44276-024-00094-2 PMC11420066

[10] Iyer K, Ren S, Pu L, et al. A graph-based approach to identify factors contributing to postoperative lung cancer recurrence among patients with non-small-cell lung cancer. Cancers. 2023;15(13):3472. 10.3390/cancers15133472. 37444581 10.3390/cancers15133472 PMC10340686

[11] Lou J, Gong B, Li Y, et al. Bone mineral density as an individual prognostic biomarker in NSCLC patients treated with immune checkpoint inhibitors. Front Immunol. 2024;15:1332303. 10.3389/fimmu.2024.1332303. 38698843 10.3389/fimmu.2024.1332303 PMC11063287

[12] Ilic I, Potthoff AL, Borger V, et al. Bone mineral density as an individual prognostic biomarker in patients with surgically-treated brain metastasis from lung cancer (NSCLC). Cancers. 2022;14(19):4633. 10.3390/cancers14194633. 36230556 10.3390/cancers14194633 PMC9562667

[13] Axelsen CT, Jensen AB, Jakobsen EH, Bechmann T. Bone loss during neoadjuvant/adjuvant chemotherapy for early stage breast cancer: a retrospective cohort study. Mol Clin Oncol. 2018;8(6):767–72. 10.3892/mco.2018.1615. 29805791 10.3892/mco.2018.1615 PMC5958697

[14] Zhang Y, Kang H, Zhao J, et al. Neoadjuvant therapy increases the risk of metabolic disorders and osteosarcopenia in patients with early breast cancer. Jpn J Clin Oncol. 2024;54(9):959–66. 10.1093/jjco/hyae070. 38807545 10.1093/jjco/hyae070

[15] Greco EA, Pietschmann P, Migliaccio S. Osteoporosis and sarcopenia increase frailty syndrome in the elderly. Front Endocrinol. 2019;10:255. 10.3389/fendo.2019.00255. 10.3389/fendo.2019.00255 PMC6491670 31068903

[16] Weilbaecher KN, Guise TA, McCauley LK. Cancer to bone: a fatal attraction. Nat Rev Cancer. 2011;11(6):411–25. 10.1038/nrc3055. 21593787 10.1038/nrc3055 PMC3666847

[17] Kanis JA. Diagnosis of osteoporosis and assessment of fracture risk. Lancet Lond Engl. 2002;359(9321):1929–36. 10.1016/S0140-6736(02)08761-5. 10.1016/S0140-6736(02)08761-5 12057569

[18] Jang S, Graffy PM, Ziemlewicz TJ, Lee SJ, Summers RM, Pickhardt PJ. Opportunistic osteoporosis screening at routine abdominal and thoracic CT: normative L1 trabecular attenuation values in more than 20 000 adults. Radiology. 2019;291(2):360–7. 10.1148/radiol.2019181648. 30912719 10.1148/radiol.2019181648 PMC6492986

[19] Gausden EB, Nwachukwu BU, Schreiber JJ, Lorich DG, Lane JM. Opportunistic use of CT imaging for osteoporosis screening and bone density assessment: a qualitative systematic review. J Bone Joint Surg Am. 2017;99(18):1580–90. 10.2106/JBJS.16.00749. 28926388 10.2106/JBJS.16.00749

[20] Pickhardt PJ, Pooler BD, Lauder T, del Rio AM, Bruce RJ, Binkley N. Opportunistic screening for osteoporosis using abdominal computed tomography scans obtained for other indications. Ann Intern Med. 2013;158(8):588–95. 10.7326/0003-4819-158-8-201304160-00003. 23588747 10.7326/0003-4819-158-8-201304160-00003 PMC3736840

[21] Hu N, Wang M, Yang M, et al. Bone mineral density in lower thoracic vertebra for osteoporosis diagnosis in older adults during CT lung cancer screening. BMC Geriatr. 2024;24(1):237. 10.1186/s12877-024-04737-4. 38448801 10.1186/s12877-024-04737-4 PMC10918915

[22] Camp RL, Dolled-Filhart M, Rimm DL. X-tile: a new bio-informatics tool for biomarker assessment and outcome-based cut-point optimization. Clin Cancer Res. 2004;10(21):7252–9. 10.1158/1078-0432.CCR-04-0713. 15534099 10.1158/1078-0432.CCR-04-0713

[23] Toshima T, Yoshizumi T, Ikegami T, et al. Impact of osteopenia in liver cirrhosis: special reference to standard bone mineral density with age. Anticancer Res. 2018;38(11):6465–71. 10.21873/anticanres.13009. 30396973 10.21873/anticanres.13009

[24] Liang W, Cai K, Chen C, et al. Expert consensus on neoadjuvant immunotherapy for non-small cell lung cancer. Transl Lung Cancer Res. 2020;9(6):2696–715. 10.21037/tlcr-2020-63. 33489828 10.21037/tlcr-2020-63 PMC7815365

[25] Ilie M, Long-Mira E, Bence C, et al. Comparative study of the PD-L1 status between surgically resected specimens and matched biopsies of NSCLC patients reveal major discordances: a potential issue for anti-PD-L1 therapeutic strategies. Ann Oncol. 2016;27(1):147–53. 10.1093/annonc/mdv489. 26483045 10.1093/annonc/mdv489

[26] Chan TA, Yarchoan M, Jaffee E, et al. Development of tumor mutation burden as an immunotherapy biomarker: utility for the oncology clinic. Ann Oncol. 2019;30(1):44–56. 10.1093/annonc/mdy495. 30395155 10.1093/annonc/mdy495 PMC6336005

[27] Dudley JC, Lin MT, Le DT, Eshleman JR. Microsatellite instability as a biomarker for PD-1 blockade. Clin Cancer Res. 2016;22(4):813–20. 10.1158/1078-0432.CCR-15-1678. 26880610 10.1158/1078-0432.CCR-15-1678

[28] Liu W, Ren S, Yang L, et al. The predictive role of hematologic markers in resectable NSCLC patients treated with neoadjuvant chemoimmunotherapy: a retrospective cohort study. Int J Surg. 2023;109(11):3519–26. 10.1097/JS9.0000000000000650. 37578441 10.1097/JS9.0000000000000650 PMC10651234

[29] Yi L, Xu Z, Ma T, et al. T-cell subsets and cytokines are indicative of neoadjuvant chemoimmunotherapy responses in NSCLC. Cancer Immunol Immunother. 2024;73(6):99. 10.1007/s00262-024-03687-5. 38619623 10.1007/s00262-024-03687-5 PMC11018727

[30] Wang F, Yang H, Chen W, et al. A combined model using pre-treatment CT radiomics and clinicopathological features of non-small cell lung cancer to predict major pathological responses after neoadjuvant chemoimmunotherapy. Curr Probl Cancer. 2024;50:101098. 10.1016/j.currproblcancer.2024.101098. 38704949 10.1016/j.currproblcancer.2024.101098

[31] Han X, Wang M, Zheng Y, et al. Delta-radiomics features for predicting the major pathological response to neoadjuvant chemoimmunotherapy in non-small cell lung cancer. Eur Radiol. 2024;34(4):2716–26. 10.1007/s00330-023-10241-x. 37736804 10.1007/s00330-023-10241-x

[32] Yang M, Li X, Cai C, et al. [18F]FDG PET-CT radiomics signature to predict pathological complete response to neoadjuvant chemoimmunotherapy in non-small cell lung cancer: a multicenter study. Eur Radiol. 2024;34(7):4352–63. 10.1007/s00330-023-10503-8. 38127071 10.1007/s00330-023-10503-8

[33] Zhuang F, Haoran E, Huang J, et al. Utility of 18F-FDG PET/CT uptake values in predicting response to neoadjuvant chemoimmunotherapy in resectable non-small cell lung cancer. Lung Cancer. 2023;178:20–7. 10.1016/j.lungcan.2023.02.001. 36764154 10.1016/j.lungcan.2023.02.001

[34] Pereira FB, Leite AF, de Paula AP. Relationship between pre-sarcopenia, sarcopenia and bone mineral density in elderly men. Arch Endocrinol Metab. 2015;59(1):59–65. 10.1590/2359-3997000000011. 25926116 10.1590/2359-3997000000011

[35] Sun K, Zuo M, Zhang Q, Wang K, Huang D, Zhang H. Anti-tumor effect of vitamin D combined with calcium on lung cancer: a systematic review and meta-analysis. Nutr Cancer. 2021;73(11–12):2633–42. 10.1080/01635581.2020.1850812. 33225749 10.1080/01635581.2020.1850812

[36] Reid IR, Bolland MJ, Grey A. Effects of vitamin D supplements on bone mineral density: a systematic review and meta-analysis. Lancet Lond Engl. 2014;383(9912):146–55. 10.1016/S0140-6736(13)61647-5. 10.1016/S0140-6736(13)61647-5 24119980

[37] Gralow JR, Biermann JS, Farooki A, et al. NCCN task force report: bone health in cancer care. J Natl Compr Cancer Netw JNCCN. 2013;11(Suppl 3):S1–50. 10.6004/jnccn.2013.0215. quiz S51. 23997241 10.6004/jnccn.2013.0215

[38] Tremblay MS, Colley RC, Saunders TJ, Healy GN, Owen N. Physiological and health implications of a sedentary lifestyle. Appl Physiol Nutr Metab. 2010;35(6):725–40. 10.1139/H10-079. 21164543 10.1139/H10-079

[39] Jones DH, Nakashima T, Sanchez OH, et al. Regulation of cancer cell migration and bone metastasis by RANKL. Nature. 2006;440(7084):692–6. 10.1038/nature04524. 16572175 10.1038/nature04524

