# Supplementary material for: Bone mineral density as a prognostic marker in patients with non-small cell lung cancer undergoing neoadjuvant chemoimmunotherapy
Source: World J Surg Oncol. 2026 Jan 8;24:69. doi: 10.1186/s12957-025-04186-2 (PMC12879409; doi:10.1186/s12957-025-04186-2)
Supplement: Supplementary file 1 — Supplementary Material 1. [file 12957_2025_4186_MOESM1_ESM.docx]

**Supplemental table 1 Operative details of all patients**

| Characteristics |  | Total |
| --- | --- | --- |
| Surgical approach | Open | 14 (13.9%) |
|  | RATS | 18 (17.8%) |
|  | VATS | 69 (68.3%) |
| Conversion to open thoracotomy | No | 100 (99.0%) |
|  | Yes | 1 (1.0%) |
| Extent of resection | Lobectomy | 91 (90.1%) |
|  | Pneumonectomy | 1 (1.0%) |
|  | Sleeve lobectomy | 9 (8.9%) |
| Postoperative hospital length of stay (Days) | Mean ± SD | 5.9 ± 3.5 |
| Estimated blood loss (ml) | Mean ± SD | 70.2 ± 48.4 |
| Atrial fibrillation | No | 88 (87.1%) |
|  | Yes | 13 (12.9%) |
| Sinus tachycardia | No | 82 (81.2%) |
|  | Yes | 19 (18.8%) |
| Chylothorax | No | 98 (97.0%) |
|  | Yes | 3 (3.0%) |
| Prolonged air leaks | No | 92 (91.1%) |
|  | Yes | 9 (8.9%) |
| Vein thrombosis | No | 95 (94.1%) |
|  | Yes | 6 (5.9%) |
| Pulmonary embolism | No | 100 (99.0%) |
|  | Yes | 1 (1.0%) |
| Bronchopleural fistula | No | 100 (99.0%) |
|  | Yes | 1 (1.0%) |

**Supplemental table 2 Pathological details of all patients**

| Characteristics |  | Total |
| --- | --- | --- |
| Histologic subtype | Non-squamous | 23 (22.8%) |
|  | Squamous | 78 (77.2%) |
| Location | Left | 36 (35.6%) |
|  | Right | 65 (64.4%) |
| MPR | No | 26 (25.7%) |
|  | Yes | 75 (74.3%) |
| pCR | No | 49 (48.5%) |
|  | Yes | 52 (51.5%) |
| pT0 | No | 48 (47.5%) |
|  | Yes | 53 (52.5%) |
| LpCR | No | 11 (10.9%) |
|  | Yes | 90 (89.1%) |
| pN | N0 | 90 (89.1%) |
|  | N1 | 2 (2.0%) |
|  | N1+N2 | 3 (3.0%) |
|  | N2 | 6 (5.9%) |
| STAS | No | 99 (98.0%) |
|  | Yes | 2 (2.0%) |
| PNI | No | 100 (99.0%) |
|  | Yes | 1 (1.0%) |
| LVI | No | 90 (89.1%) |
|  | Yes | 11 (10.9%) |
| TE | No | 100 (99.0%) |
|  | Yes | 1 (1.0%) |
| VPI | No | 98 (97.0%) |
|  | Yes | 3 (3.0%) |

MPR, major pathologic response; pCR, pathological complete response; pT0, pathologic T0 stage; LpCR, lymph node pathologic complete response; pN, pathologic N stage; STAS, spread through air spaces; PNI, perineural invasion; LVI, lympho-vascular invasion; TE, tumor embolism; VPI, visceral pleural invasion

**Supplemental table 3 Bone mineral density parameters of the patients**

| Characteristics |  | Non-MPR (N=26) | MPR (N=75) | p |
| --- | --- | --- | --- | --- |
| Baseline T10 BMD (HU) | Mean ± SD | 157.3 ± 63.3 | 175.6 ± 42.7 | 0.179 |
| Baseline T12 BMD (HU) | Mean ± SD | 137.9 ± 54.4 | 157.7 ± 42.0 | 0.059 |
| Baseline L1 BMD (HU) | Mean ± SD | 133.2 ± 55.1 | 149.3 ± 42.9 | 0.131 |
| Preoperative T10 BMD (HU) | Mean ± SD | 142.0 ± 47.3 | 161.3 ± 43.5 | 0.06 |
| Preoperative T12 BMD (HU) | Mean ± SD | 126.6 ± 50.7 | 145.7 ± 48.1 | 0.087 |
| Preoperative L1 BMD (HU) | Mean ± SD | 121.0 ± 50.6 | 137.2 ± 46.6 | 0.139 |
| dT10 (HU) | Mean ± SD | 15.3 ± 35.6 | 14.3 ± 31.7 | 0.901 |
| dT12 (HU) | Mean ± SD | 11.4 ± 31.6 | 12.0 ± 25.0 | 0.923 |
| dL1 (HU) | Mean ± SD | 12.2 ± 31.8 | 12.1 ± 24.3 | 0.98 |
| Baseline L1 BMD (by best cut-off) | Low | 14 (53.8%) | 31 (41.3%) | 0.38 |
|  | High | 12 (46.2%) | 44 (58.7%) |  |
| Preoperative L1 BMD (by best cut-off) | Low | 17 (65.4%) | 35 (46.7%) | 0.156 |
|  | High | 9 (34.6%) | 40 (53.3%) |  |
| dL1 BMD (by best cut-off) | Low | 12 (46.2%) | 36 (48%) | 1 |
|  | High | 14 (53.8%) | 39 (52%) |  |
| Baseline age-adjust BMD | Low | 19 (73.1%) | 53 (70.7%) | 1 |
|  | High | 7 (26.9%) | 22 (29.3%) |  |
| Preoperative age-adjust BMD | Low | 20 (76.9%) | 52 (69.3%) | 0.627 |
|  | High | 6 (23.1%) | 23 (30.7%) |  |

BMD, bone mineral density; HU, Hounsfield unit

**Supplemental table 4 Subgroup cox regression analysis of baseline L1 BMD (per SD) on DFS**

| Characteristics | Count | Percent | HR (95% CI) | P value | P for interaction |
| --- | --- | --- | --- | --- | --- |
| Overall | 101 | 100 | 0.47(0.25 to 0.86) | **0.014** |  |
| Age (years) |  |  |  |  | 0.076 |
| <65 | 71 | 70.3 | 0.31(0.12 to 0.80) | **0.016** |  |
| ≥65 | 30 | 29.7 | 0.88(0.34 to 2.30) | 0.791 |  |
| Smoke |  |  |  |  | 0.795 |
| Former or current | 71 | 70.3 | 0.51(0.25 to 1.03) | 0.06 |  |
| Never | 30 | 29.7 | 0.29(0.06 to 1.28) | 0.102 |  |
| BMI (Kg/m2) |  |  |  |  | 0.579 |
| <25 | 54 | 53.5 | 0.29(0.10 to 0.87) | **0.028** |  |
| ≥25 | 47 | 46.5 | 0.54(0.23 to 1.26) | 0.155 |  |
| Clinical stage |  |  |  |  | 0.183 |
| IIA-B | 24 | 23.8 | 1.00(1.00 to 1.00) |  |  |
| IIIA | 41 | 40.6 | 0.33(0.14 to 0.82) | **0.017** |  |
| IIIB | 36 | 35.6 | 0.62(0.28 to 1.39) | 0.246 |  |
| Histologic subtype |  |  |  |  | 0.706 |
| Non-squamous | 23 | 22.8 | 0.49(0.16 to 1.46) | 0.199 |  |
| Squamous | 78 | 77.2 | 0.35(0.14 to 0.87) | **0.023** |  |
| Location |  |  |  |  | 0.219 |
| Left | 36 | 35.6 | 0.78(0.31 to 2.02) | 0.615 |  |
| Right | 65 | 64.4 | 0.28(0.11 to 0.75) | 0.012 |  |
| Dosage |  |  |  |  | 0.426 |
| 2 | 25 | 24.8 | 0.70(0.25 to 1.93) | 0.491 |  |
| 3 | 40 | 39.6 | 0.30(0.09 to 0.97) | **0.044** |  |
| 4 | 36 | 35.6 | 0.49(0.17 to 1.40) | 0.182 |  |
| Interval (weeks) |  |  |  |  | 0.917 |
| >6 | 55 | 54.5 | 0.53(0.24 to 1.18) | 0.119 |  |
| ≤6 | 46 | 45.5 | 0.41(0.14 to 1.20) | 0.104 |  |
| MPR |  |  |  |  | **0.007** |
| No | 26 | 25.7 | 1.06(0.57 to 1.97) | 0.865 |  |
| Yes | 75 | 74.3 | 0.16(0.05 to 0.53) | **0.003** |  |
| pCR |  |  |  |  | 0.279 |
| No | 50 | 49.5 | 0.71(0.36 to 1.39) | 0.32 |  |
| Yes | 51 | 50.5 | 0.18(0.04 to 0.83) | **0.028** |  |

**Supplemental table 5 Subgroup cox regression analysis of preoperative L1 BMD (per SD) on DFS**

| Characteristics | Count | Percent | HR (95% CI) | P value | P for interaction |
| --- | --- | --- | --- | --- | --- |
| Overall | 101 | 100 | 0.53(0.29 to 0.98) | **0.041** |  |
| Age (years) |  |  |  |  | **0.002** |
| <65 | 71 | 70.3 | 0.19(0.06 to 0.63) | **0.007** |  |
| ≥65 | 30 | 29.7 | 1.57(0.60 to 4.09) | 0.354 |  |
| Smoke |  |  |  |  | 0.236 |
| Former or current | 71 | 70.3 | 0.46(0.22 to 0.95) | **0.036** |  |
| Never | 30 | 29.7 | 0.96(0.37 to 2.50) | 0.926 |  |
| BMI (Kg/m2) |  |  |  |  | 0.861 |
| <25 | 54 | 53.5 | 0.43(0.16 to 1.15) | 0.093 |  |
| ≥25 | 47 | 46.5 | 0.51(0.21 to 1.28) | 0.152 |  |
| Clinical stage |  |  |  |  | 0.231 |
| IIA-B | 24 | 23.8 | 1.00(1.00 to 1.00) |  |  |
| IIIA | 41 | 40.6 | 0.61(0.27 to 1.38) | 0.235 |  |
| IIIB | 36 | 35.6 | 0.44(0.18 to 1.07) | 0.071 |  |
| Histologic subtype |  |  |  |  | 0.699 |
| Non-squamous | 23 | 22.8 | 0.63(0.25 to 1.61) | 0.339 |  |
| Squamous | 78 | 77.2 | 0.40(0.16 to 0.99) | **0.047** |  |
| Location |  |  |  |  | 0.86 |
| Left | 36 | 35.6 | 0.51(0.17 to 1.57) | 0.244 |  |
| Right | 65 | 64.4 | 0.52(0.24 to 1.14) | 0.103 |  |
| Dosage |  |  |  |  | 0.8 |
| 2 | 25 | 24.8 | 0.49(0.15 to 1.56) | 0.227 |  |
| 3 | 40 | 39.6 | 0.61(0.25 to 1.51) | 0.283 |  |
| 4 | 36 | 35.6 | 0.43(0.14 to 1.28) | 0.129 |  |
| Interval (weeks) |  |  |  |  | 0.158 |
| >6 | 55 | 54.5 | 0.38(0.16 to 0.92) | **0.032** |  |
| ≤6 | 46 | 45.5 | 0.82(0.34 to 1.98) | 0.662 |  |
| MPR |  |  |  |  | 0.06 |
| No | 26 | 25.7 | 1.05(0.50 to 2.20) | 0.893 |  |
| Yes | 75 | 74.3 | 0.29(0.11 to 0.77) | **0.014** |  |
| pCR |  |  |  |  | 0.831 |
| No | 50 | 49.5 | 0.66(0.32 to 1.35) | 0.254 |  |
| Yes | 51 | 50.5 | 0.45(0.14 to 1.39) | 0.164 |  |

**Supplemental table 6 Sensitive analysis of BMD (per SD) on DFS**

| Characteristics | HR (95% CI) | P value |
| --- | --- | --- |
| Baseline T10 BMD (per SD) | 0.689(0.398,1.190) | 0.184 |
| Preoperative T10 BMD (per SD) | 0.704(0.418,1.190) | 0.187 |
| dT10 BMD (per SD) | 0.960(0.585,1.570) | 0.87 |
| Baseline T12 BMD (per SD) | 0.553(0.307,0.996) | **0.049** |
| Preoperative T12 BMD (per SD) | 0.577(0.323,1.030) | 0.064 |
| dT12 BMD (per SD) | 0.974(0.590,1.610) | 0.918 |
| Baseline L1 BMD (per SD) | 0.465(0.252,0.859) | **0.015** |
| Preoperative L1 BMD (per SD) | 0.529(0.286,0.976) | **0.042** |
| dL1 BMD (per SD) | 0.829(0.500,1.370) | 0.466 |

**Supplemental table 7 Uni-variable and multi-variable logistic regression on pCR**

| Characteristics |  | OR (univariable) | OR (multivariable) |
| --- | --- | --- | --- |
| Age (years) | <65 |  |  |
|  | ≥65 | 0.66 (0.28-1.57, p=.351) |  |
| Gender | Female |  |  |
|  | Male | 0.49 (0.09-2.80, p=.422) |  |
| Smoke | Former or current | |  |
|  | Never | 1.18 (0.50-2.76, p=.711) |  |
| BMI (Kg/m²） | <25 |  |  |
|  | ≥25 | 1.44 (0.65-3.15, p=.366) |  |
| Clinical stage | IIA-B |  |  |
|  | IIIA | 0.81 (0.29-2.21, p=.675) |  |
|  | IIIB | 0.85 (0.30-2.38, p=.752) |  |
| cT | T1 |  |  |
|  | T2 | 1.12 (0.28-4.54, p=.876) |  |
|  | T3 | 0.65 (0.15-2.77, p=.557) |  |
|  | T4 | 1.45 (0.34-6.25, p=.615) |  |
| cN | N0 |  |  |
|  | N1 | 2.00 (0.53-7.54, p=.306) |  |
|  | N2-3 | 1.25 (0.39-4.03, p=.709) |  |
| Histologic subtype | Non-squamous | |  |
|  | Squamous | 2.96 (1.09-8.00, p=**.033**) | 3.38 (1.09-10.47, p=**.035**) |
| Location | Left |  |  |
|  | Right | 1.23 (0.54-2.77, p=.625) |  |
| PD-L1 (TPS) | <1% |  |  |
|  | >50% | 1.61 (0.37-6.92, p=.524) |  |
|  | 1-50% | 1.66 (0.42-6.50, p=.466) |  |
|  | Not known | 0.75 (0.05-11.31, p=.835) |  |
| Chemotherapy | ——^*1^ |  |  |
| Immunotherapy | ——^*1^ |  |  |
| Dosage | 2 |  |  |
|  | 3 | 1.56 (0.57-4.25, p=.389) |  |
|  | 4 | 1.27 (0.46-3.55, p=.645) |  |
| Interval (weeks) | >6 week |  |  |
|  | ≤6 week | 1.84 (0.83-4.06, p=.133) | 2.36 (0.95-5.84, p=.064) |
| Lesion size (mm) | Mean ± SD | 0.96 (0.93-0.99, p=**.016**) | 0.97 (0.94-1.00, p=**.048**) |
| pN | ——^*1^ |  |  |
| Baseline serum Ca (mmol/L) | Mean ± SD | 1.53 (0.17-13.67, p=.704) |  |
| Baseline A/G | Mean ± SD | 1.44 (0.31-6.75, p=.642) |  |
| Preoperative serum Ca (mmol/L) | Mean ± SD | 1.26 (0.38-4.21, p=.704) |  |
| Preoperative A/G | Mean ± SD | 2.04 (0.43-9.61, p=.368) |  |
| Baseline L1 BMD (HU) | Mean ± SD | 1.00 (1.00-1.01, p=.399) |  |
| Preoperative L1 BMD (HU) | Mean ± SD | 1.01 (1.00-1.02, p=.122) |  |
| dL1 BMD (HU) | Mean ± SD | 0.99 (0.97-1.00, p=.181) |  |
| Baseline L1 BMD | Low |  |  |
|  | High | 1.32 (0.60-2.90, p=.491) |  |
| Preoperative L1 BMD | Low |  |  |
|  | High | 1.68 (0.77-3.69, p=.196) | 1.29 (0.54-3.07, p=.561) |
| dL1 BMD | Low |  |  |
|  | High | 0.64 (0.29-1.41, p=.272) |  |

^*1^ Due to the small sample size of some subgroups, chemotherapy, immunotherapy, and pN were excluded from the logistic regression analysis.

A/G, albumin to globulin ratio; BMD, bone mineral density; HU, Hounsfield unit; MPR, major pathologic response; pCR, pathologic complete response; pN, pathologic N stage; SD, standard deviation


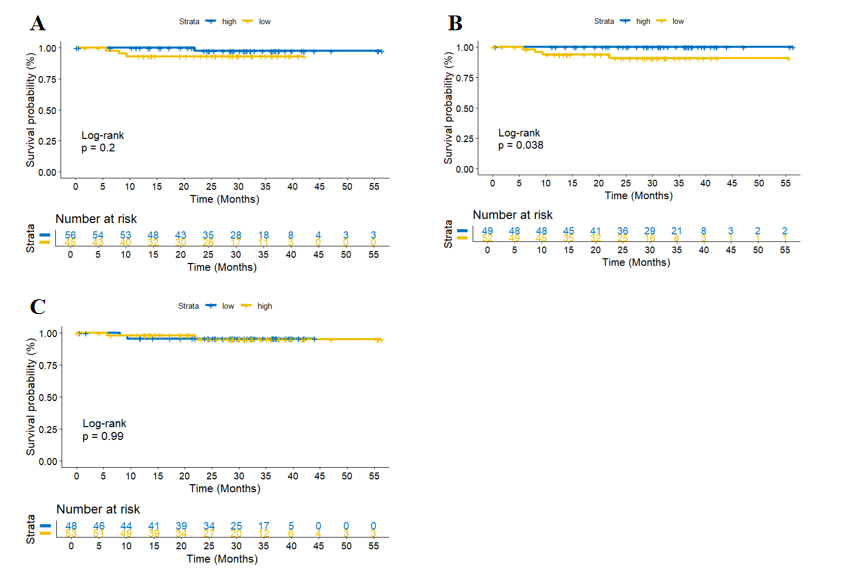


**Supplemental Figure 1. Association of baseline, preoperative, and dL1 BMD with overall survival (OS).** Kaplan-Meier curves of OS are shown stratified by **(A)** baseline BMD, **(B)** preoperative BMD, and **(C)** dL1 BMD. Patients were divided into high and low groups based on optimal cutoff values of 132 HU, 124.2 HU, and 9.73 HU, respectively.

**
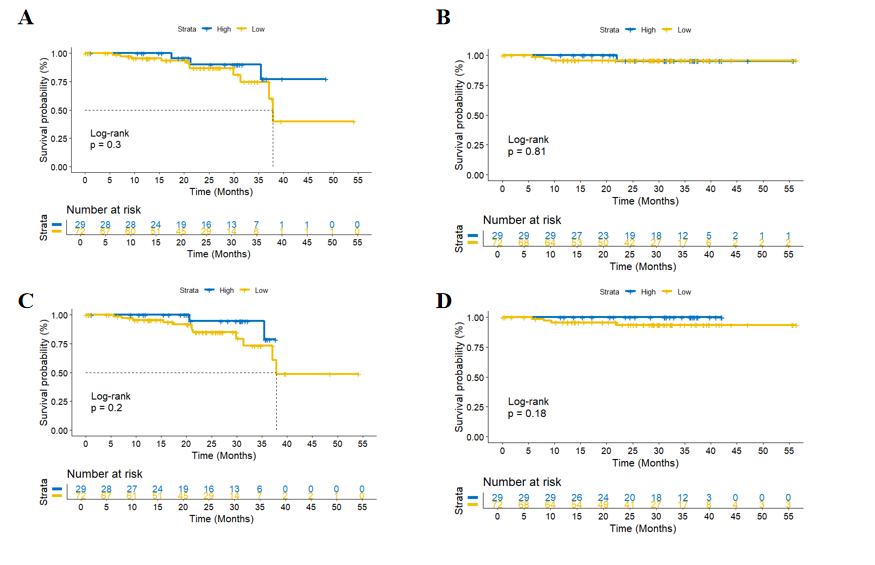
**

**Supplemental Figure 2. Association of age-adjusted BMD with disease-free survival (DFS) and overall survival (OS).** Kaplan-Meier curves illustrate **(A)** DFS and **(B)** OS stratified by baseline BMD, as well as **(C)** DFS and **(D)** OS stratified by preoperative BMD. The blue and yellow lines represent the high and low BMD groups, respectively.
